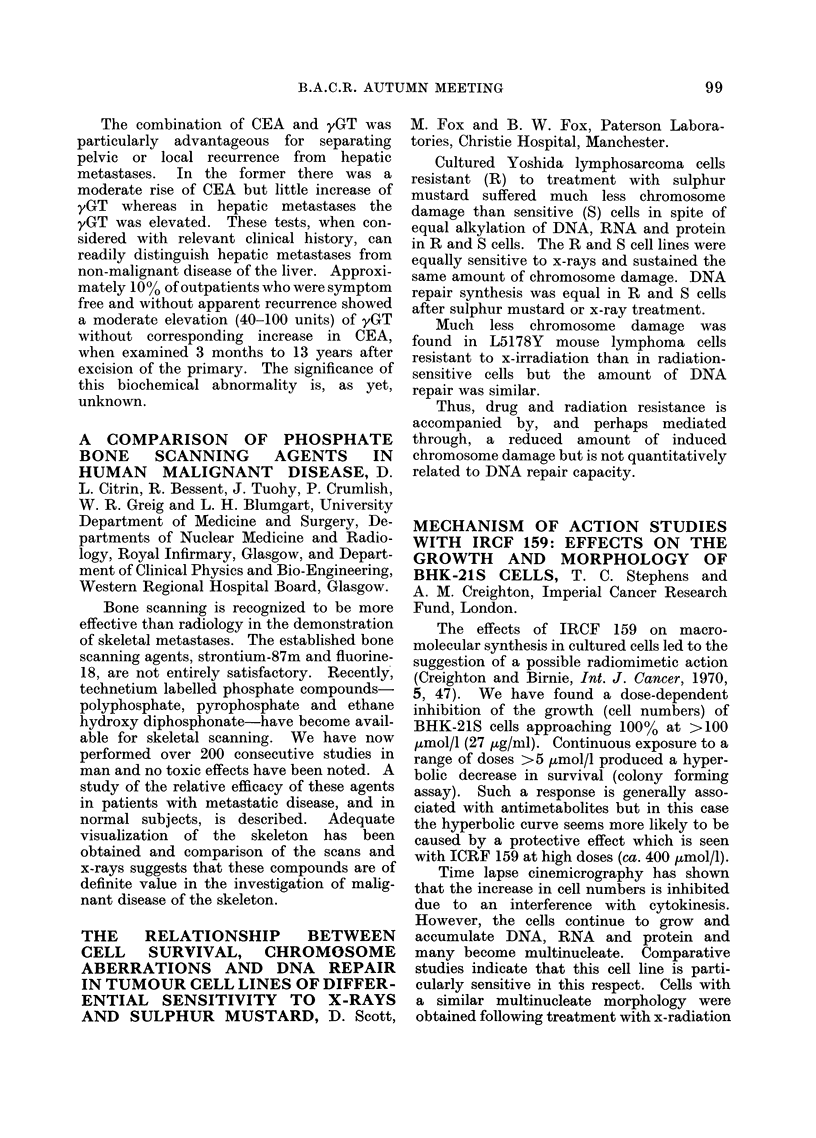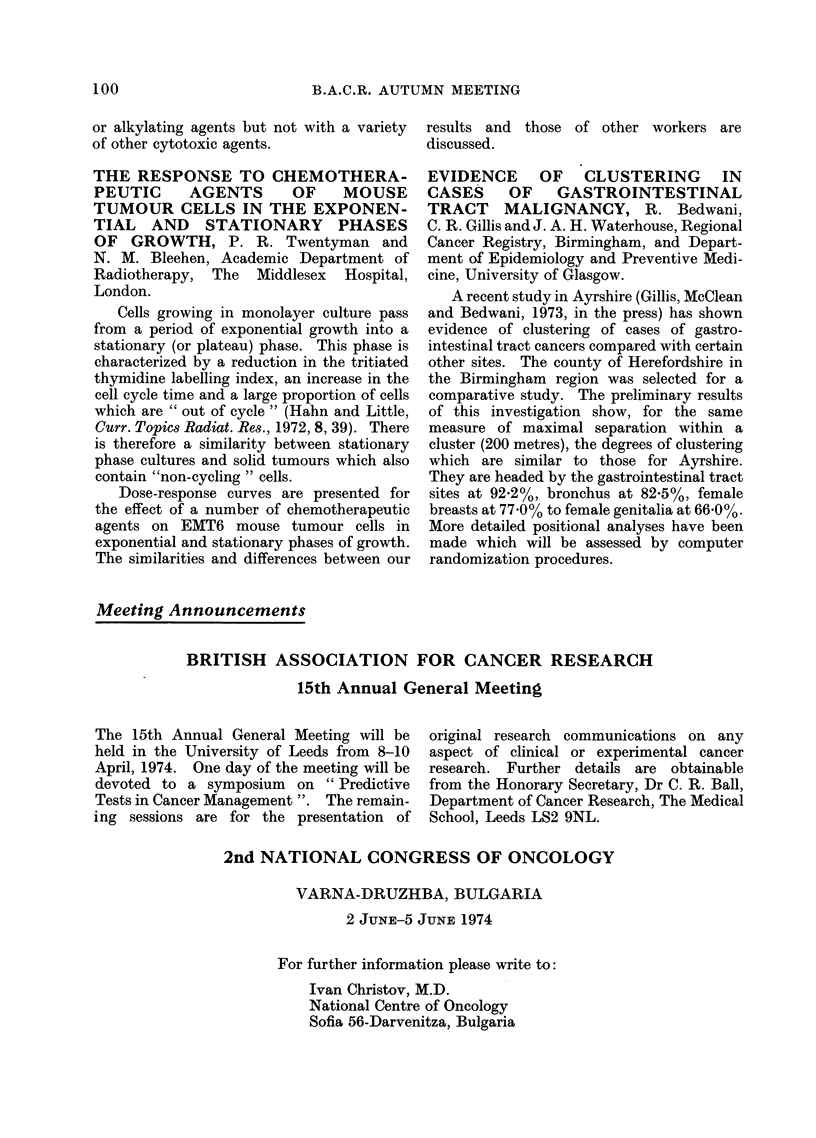# Proceedings: Mechanism of action studies with IRCF 159: effects on the growth and morphology of BHK-21S cells.

**DOI:** 10.1038/bjc.1974.39

**Published:** 1974-01

**Authors:** T. C. Stephens, A. M. Creighton


**MECHANISM OF ACTION STUDIES WITH IRCF 159: EFFECTS ON THE GROWTH AND MORPHOLOGY OF BHK-21S CELLS,** T. C. Stephens and A. M. Creighton, Imperial Cancer Research Fund, London.

The effects of IRCF 159 on macromolecular synthesis in cultured cells led to the suggestion of a possible radiomimetic action (Creighton and Birnie, *Int. J. Cancer*, 1970, **5**, 47). We have found a dose-dependent inhibition of the growth (cell numbers) of BHK-21S cells approaching 100% at >100 μmol/l (27 μg/ml). Continuous exposure to a range of doses >5 μmol/l produced a hyperbolic decrease in survival (colony forming assay). Such a response is generally associated with antimetabolites but in this case the hyperbolic curve seems more likely to be caused by a protective effect which is seen with ICRF 159 at high doses (*ca.* 400 μmol/l).

Time lapse cinemicrography has shown that the increase in cell numbers is inhibited due to an interference with cytokinesis. However, the cells continue to grow and accumulate DNA, RNA and protein and many become multinucleate. Comparative studies indicate that this cell line is particularly sensitive in this respect. Cells with a similar multinucleate morphology were obtained following treatment with x-radiation or alkylating agents but not with a variety of other cytotoxic agents.